# Sensitivity Analysis for Predicting Sub-Micron Aerosol Concentrations Based on Meteorological Parameters

**DOI:** 10.3390/s20102876

**Published:** 2020-05-19

**Authors:** Martha A. Zaidan, Ola Surakhi, Pak Lun Fung, Tareq Hussein

**Affiliations:** 1 Institute for Atmospheric and Earth System Research (INAR)/Physics, University of Helsinki, FI-00560 Helsinki, Finland; pak.fung@helsinki.fi; 2 Department of Computer Science, The University of Jordan, Amman 11942, Jordan; ola.surakhi@gmail.com; 3 Department of Physics, The University of Jordan, Amman 11942, Jordan

**Keywords:** particle number concentration, modeling, sensitivity analysis, artificial neural networks, feed-forward neural network, time-delay neural network

## Abstract

Sub-micron aerosols are a vital air pollutant to be measured because they pose health effects. These particles are quantified as particle number concentration (PN). However, PN measurements are not always available in air quality measurement stations, leading to data scarcity. In order to compensate this, PN modeling needs to be developed. This paper presents a PN modeling framework using sensitivity analysis tested on a one year aerosol measurement campaign conducted in Amman, Jordan. The method prepares a set of different combinations of all measured meteorological parameters to be descriptors of PN concentration. In this case, we resort to artificial neural networks in the forms of a feed-forward neural network (FFNN) and a time-delay neural network (TDNN) as modeling tools, and then, we attempt to find the best descriptors using all these combinations as model inputs. The best modeling tools are FFNN for daily averaged data (with R${}^{2}=0.77$) and TDNN for hourly averaged data (with R${}^{2}=0.66$) where the best combinations of meteorological parameters are found to be temperature, relative humidity, pressure, and wind speed. As the models follow the patterns of diurnal cycles well, the results are considered to be satisfactory. When PN measurements are not directly available or there are massive missing PN concentration data, PN models can be used to estimate PN concentration using available measured meteorological parameters.

## 1. Introduction

### 1.1. Motivation

Approximately seven million people die every year due to adverse health-related air pollution issues, in which 4.2 million deaths are attributed to exposure to poor outdoor air quality. Approximately 91% of the world’s population lives in areas where air pollution exceeds guideline limits established by the World Health Organization (WHO) [1]. The most critical air pollutants from a health perspective include airborne particulate matter (PM) and the gaseous pollutants, such as ozone (O${}_{3}$), nitrogen dioxide (NO${}_{2}$), volatile organic compounds (e.g., benzene), carbon monoxide (CO), and sulfur dioxide (SO${}_{2}$) [2,3]. In particular, particles less than 2.5 micrometers in diameter (PM${}_{2.5}$) are able to penetrate deeply into human lungs, irritate and corrode the alveolar wall, and consequently impair lung function. Although the diameter of PM${}_{2.5}$ is very small, it has a large surface area, and then may be capable of carrying various toxic substances, passing through the filtration of nose hair, reaching the end of the respiratory tract with airflow, and accumulating there by diffusion, damaging other parts of the body through air exchange in the lungs [4]. Atmospheric PM also plays a role in ecosystems and Earth’s climate, leading to extensive research on the subject [5].

A critically important class of atmospheric PM is called ultra-fine particles (UFPs). These particles are smaller than 100 nm in size (i.e., sub-micron aerosols). Scientific attention has recently moved toward UFPs because these particles have very high surface area to mass ratios. Consequently, they can easily enter the human respiratory system and deposit preferentially in the deepest areas of the lungs, such as the tracheobronchial and alveolar regions, carrying toxic compounds [6]. Emissions associated with traffic, industrial activities, and domestic heating contribute to a large fraction of UFPs [7]. Particle number (PN) concentrations are more informative in describing the abundance of UFPs because these particles tend to dominate atmospheric PM number size distributions and contribute little to PM mass concentrations that are presently used as air quality indicators (e.g., PM${}_{2.5}$ and PM${}_{10}$) [8]. Unfortunately, there are much fewer data available on PN compared with PM [9,10] due to the unavailability of instruments for measuring UFP in many air quality monitoring stations [6]. Therefore, we propose in this paper a modeling framework to be an alternative method in estimating PN concentration using other available measurements. In this way, PN concentration can be monitored in cities where the measurements are not available, and the air quality database can be updated for further analysis.

### 1.2. Data-Driven Air Pollutant Modeling

Modeling air pollutants can generally be categorized into three main approaches, including: physics- and expert-based and data-driven approaches [11]. First, physics-based approaches use models that describe underlying physical processes related to air pollutants directly [12]. This modeling approach is typically accurate and reliable, but physics and chemistry knowledge is required, especially related to a particular air pollutant to be modeled. In some cases, they can be computationally demanding and may also be sensitive to the scale and quality of the parameters involved [13]. Examples are the urban airshed model (UAM) [14] and the community multiscale air quality (CMAQ) model [15]. Second, expert-based approaches, such as the expert elicitation process [16], elicit knowledge from experts/specialists for modeling and analysis [17]. The involvement of experts may be helpful to explain data anomalies or pattern outliers due to untypical air pollution phenomena, such as forest fires, sudden traffic changes, etc. However, it is often difficult to find agreement among experts about the use of expert systems and how the uncertainties of different variables can be adequately accounted for [16]. Finally, the data-driven approach uses historical datasets to identify relationships between measured variables and then builds models based on the trends in the data. This approach does not typically require deep knowledge in air pollutant dynamics, chemistry composition, and other explanatory variables. Due to these reasons, more practitioners have recently utilized data-driven approaches, such as neural networks, as alternatives to physics- and expert-based methods, to model air pollutant concentrations [18]. This work resorts to a data-driven approach in the form of artificial neural networks (ANN) to model and estimate PN concentrations. In particular, sensitivity analysis is carried out to find the best combination of measured variables for estimating PN concentrations.

Data-driven-based modeling has been carried out for estimating different air pollutant concentrations, including nitrogen dioxide (NO${}_{2}$) [19], sulfur dioxide (SO${}_{2}$) [20,21], ozone (O${}_{3}$) [22,23], black carbon [11,24], particulate matter smaller than 10 μm (PM${}_{10}$) [21,25], and particulate matter smaller than 2.5 μm (PM${}_{2.5}$) [26,27,28]. However, there is a very limited number of studies focusing on estimating PN concentration. The estimations of PN concentration using data-driven methods were focused on European cities, described in [29,30]. For the first time, this work proposes a data-driven framework for estimating PN concentration in the Middle East and North Africa (MENA) region. Furthermore, the modeling framework evaluates the performance by applying different combination of measured variables, which is known as sensitivity analysis. The best combination of measured variables leads to reliable PN models and then allows filling in the missing data in the air quality database and estimating the PN concentration without relying on expensive measurements. The capability to estimate PN concentration on a daily and hourly basis allows a decision maker, such as a government agency, to mitigate the impact caused from these sub-micron aerosols.

## 2. Materials

This section describes the materials used in this study. We explain the experimental setup, and then, we describe how the data were pre-processed. We also discuss the environmental conditions during the measurement period.

### 2.1. Database

In this study, we used a database obtained from a measurement campaign in Amman, the capital city of Jordan, from 1 August 2016 until 31 July 2017. The city is considered as an area with Middle Eastern urban conditions within the MENA region. This region serves as a compilation of different aerosol particle sources including natural dust, anthropogenic pollution (e.g., generated from the petrochemical industry and urbanization), as well as new particle formation [31].

The database includes sub-micron particle number concentration (PN) and meteorological conditions. The aerosol measurement was performed at the aerosol laboratory, which is located on the third floor of the Department of Physics, University of Jordan. The campus is located in an urban background in the north part of Amman, Jordan. In particular, the campaign measured the particle number size distribution using a scanning mobility particle sizer (NanoScan SMPS 3910, TSI, MN, USA). The time resolution used in the SMPS was 1 min. The meteorological measurement was performed with a weather station (WH-1080, Clas Ohlson: Art.no.36-3242, Helsinki, Finland) with a 5 min time resolution. The meteorological data were comprised of ambient temperature (T), absolute pressure (P), relative humidity (RH), wind speed (WS), and wind direction (WD). The details of the aerosol measurement campaign and the meteorological measurements were described in [31,32].

### 2.2. Data Handling

The particle number concentrations (PN), in cm${}^{-3}$, were calculated by integrating the measured particle number size distribution over the specified particle diameter range, given by:

(1) $${\mathrm{PN}}_{\mathrm{sub}}=\int_{10\;\mathrm{nm}}^{450\;\mathrm{nm}}n_{N}^{0}\;dlog_{10}\left(Dp\right)$$

where $n_{N}^{0}=dN/dlog_{10}\left(Dp\right)$ is the measured particle number size distribution and Dp is the particle diameter. Since air quality data are typically reported hourly or daily, the processed aerosol data (${\mathrm{PN}}_{\mathrm{sub}}$ concentration) and meteorological measurements were averaged hourly and daily. Having the data for a year at an hourly resolution allowed the modeling to capture the diurnal cycle and seasonal variability.

### 2.3. Environmental Conditions

**Figure 1:**
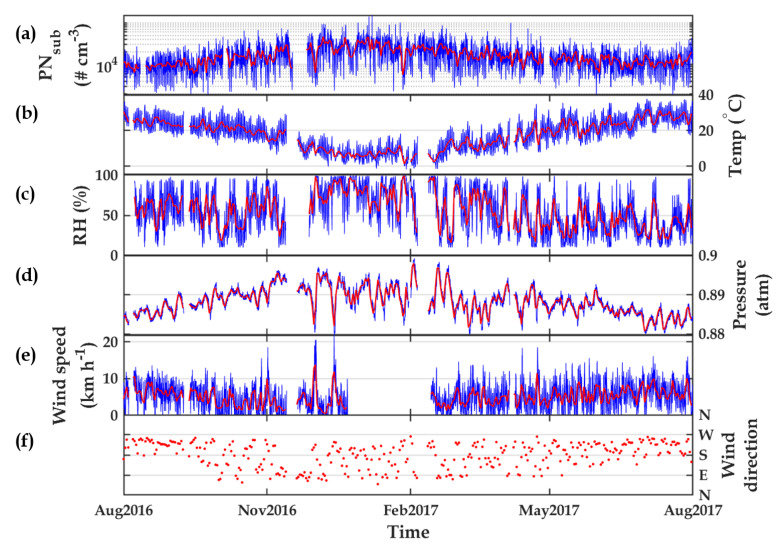
PNsub, shown in subfigure (**a**), and meteorological conditions, shown in subfigures (**b**–**f**), during the experiments. Red and blue colors are daily and hourly averaged.

**Figure 2:**
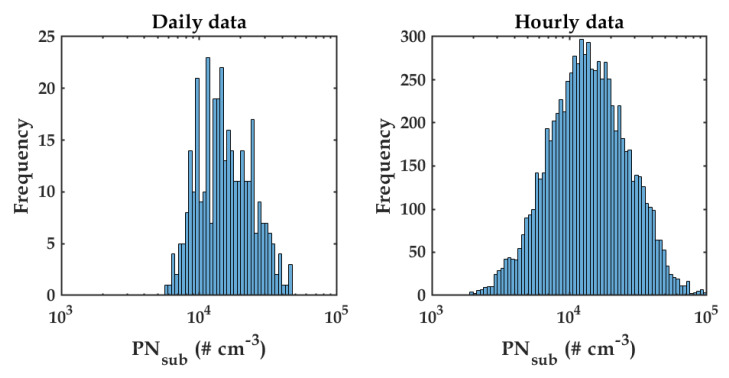
PN number concentration histograms during the experiments for daily averaged data (**left**) and hourly averaged data (**right**).

Figure 1a shows time-series data of PN during the campaign. The red curve represents the daily average, whereas the blue curve indicates the hourly measurement. It can be seen that the PN concentration ranged between 10${}^{3}$ cm${}^{-3}$ and 10${}^{5}$ cm${}^{-3}$, with median values of 1400 cm${}^{-3}$ and 1500 cm${}^{-3}$, for daily and hourly data, respectively. In addition, Figure 2 presents PN histograms for daily (left subplot) and hourly (right subplot). It can be seen that both histograms peaked at the bin edge at about 1330 cm${}^{-3}$. Understanding the ranges and the median values of PN${}_{\mathrm{sub}}$ concentrations allowed us to examine later if the modeling metrics were adequate.

Figure 1b–f present the meteorological conditions during the experiment. The red and blue curves indicate daily and hourly averaged data. Figure 1b shows the temperature (T), with a minimum peak of about 0 ${}^{{}^{\circ}}$C during winter and a maximum of about 40 ${}^{{}^{\circ}}$C during summer with the hourly median value of 19.9 ${}^{{}^{\circ}}$C. Figure 1c indicates the relative humidity (RH), which varied between 10% and 100% with the hourly median value of 52.3%. Figure 1d is the pressure (P), which ranged between 0.88 atm and 0.9 atm, and its median value was 0.888 atm. Figure 1e indicates wind speed (WS), ranging between 0 km/h and 20 km/h with a median value of 5 km/h. It can be seen that there were about 2 months of missing data in this variable. Finally, Figure 1f represents wind direction (WD), where only daily averaged data are shown for better visualization. Wind blows mainly from the south and west (180${}^{{}^{\circ}}$–270${}^{{}^{\circ}}$) from June to September. The wind direction varies in other months, ranging from 45${}^{{}^{\circ}}$ to 270${}^{{}^{\circ}}$.

## 3. Methods

**Figure 3:**
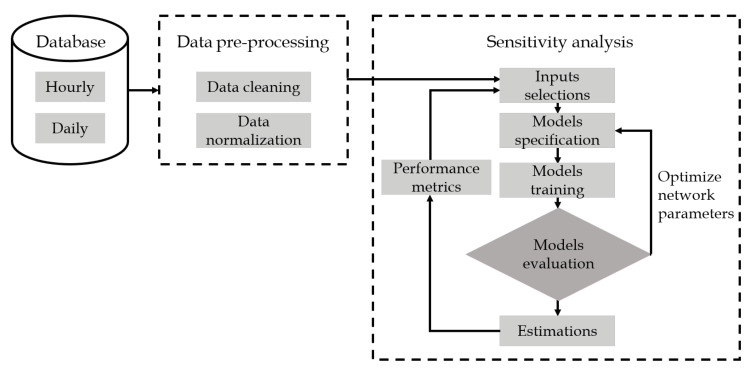
The block diagram of the proposed sensitivity analysis for estimating PN concentration.

This section describes the methodology for estimating PN concentration used in this study. Figure 3 shows a block diagram illustrating this methodology. First, a database was formed using processed data from aerosol and meteorological measurements as described in Section 2.2. In the second step, the data underwent pre-processing procedures through data cleaning and data normalization, which will be explained in Section 3.1. The next step was a part of the sensitivity analysis block, consisting of several sub-steps.

In this work, sensitivity analysis could be defined as a methodology to find the best combination of measured variables for modeling PN concentration. Sensitivity analysis is more effective than performing bivariate correlation analysis, including linear correlation, such as Pearson [33] and Spearman [34], and non-linear correlation analysis, such as mutual information [35]. Bivariate correlation analysis is beneficial when two variables are investigated in terms of their relationship, but when there are more than two variables interacting in multivariate directions, those methods may no longer be effective. The first sub-step in sensitivity analysis is input selection. This sub-step prepares a set of different measured variable combinations. Every single combination is then fed as inputs for a chosen data-driven model. The next sub-step is to specify the chosen model structure and other model properties. Then, the model parameters can be optimized in the model training sub-step. Once the model parameters have been optimized, the model is then evaluated using selected metrics; if the performance is not satisfactory, the model structure needs to be re-specified. These steps are done iteratively until we achieve satisfactory performance defined by a modeler. Once the model has met satisfactory condition, it estimates PN concentration using test data. Finally, performance metrics can be evaluated, and the next sub-step is to take other input combinations. These sub-steps can be done in sequence or in parallel depending on available computing resources.

### 3.1. Data Pre-Processing

The aerosol measurement data obtained from 1 August 2016 to 31 July 2017 were processed to give the PN concentration. The meteorological measurements (T, RH, P, WS, WD) were collected for the same period. Both data were merged, and the data were averaged daily and hourly, resulting in 365 and 8760 observations, respectively. The data pre-processing began by removing the missing data. The missing data constituted 6.7% of the total data points due to technical faults or instrument maintenance. Since the data were obtained from different measured variables with various physical units and magnitude, it was crucial to normalize the data. The scaling factor depended on the chosen model, which contained activation functions ranging between these values. In this case, the data were scaled between 0 and 1 to transform them into the range of the activation function.

### 3.2. Modeling

**Figure 4:**
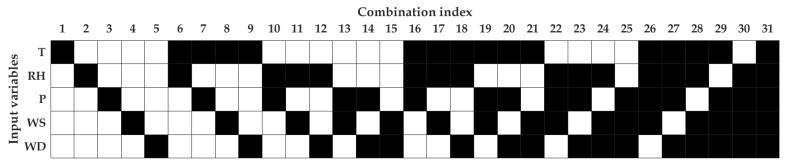
Sensitivity analysis uses different combinations of meteorological variables as inputs for PN modeling.

The first sub-step in sensitivity analysis is to prepare a set of measured variable combinations, as shown in Figure 4. Then, the inputs for a chosen model are selected based on this combination, which can be done in sequence or in parallel.

The second sub-step is to prepare a PN model. In this case, we used an artificial neural network (ANN) to model PN concentration. Neural networks (NNs) provide a robust approach for approximating real-valued (regression) and discrete-valued (classification) target functions because they can mimic the non-linearity of the functions and their optimization methods are well developed [36]. These models have been a popular choice among machine learning methods for approximating complex functions [37] and have been utilized in a large number of applications [38], including air pollution [18] and climate [39]. In this case, we resorted to two types of NNs, which were a feed-forward neural network (FFNN) and a time-delay neural network (TDNN). FFNN is a fully-connected network with two layers (input and hidden layers). FFNN has been the mostly popular choice of NNs due to its fast operation, ease of implementation, and smaller training set requirements [40]. TDNN structure is the same as FFNN, but the feed-forward network has a tapped delay line at the input. TDNN is part of a general class of dynamic networks, where the dynamics appear only at the input layer of a static multi-layer feed-forward network. This type of network is suited well for dealing with time-series data [41].

**Figure 5:**
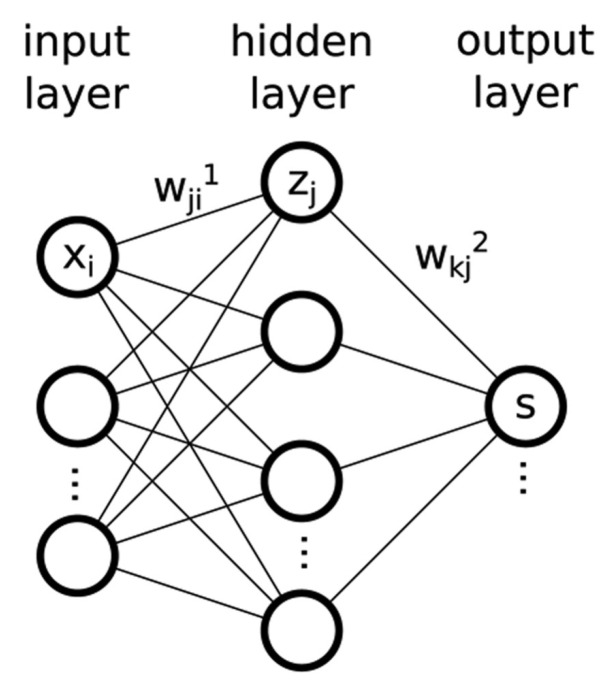
Schematic representation of a neural network with one hidden layer.

Both FFNN and TDNN estimate PN concentration, $\hat{y}$, through the function of meteorological variables, f(x,w), by optimizing the weights, w, of NN. Figure 5 displays a schematic representation of a neural network with one hidden layer. The *j*th neuron in the *L*th layer calculates the output $z_{j}^{L}$ as:

(2) $$z_{j}^{L}=\sigma \left(\sum_{i}w_{ji}^{L}\;x_{i}+b_{j}^{L}\right)$$

where the notation $w_{ji}^{L}$ represents the weight of connection between the computing neuron and its *i*th input in the preceding layer and $b_{j}^{L}$ is a bias parameter. In the case of TDNN, a tapped delay line is introduced at the input layer, where the input data are buffered for several time steps and then fed to the input layer. The introduction of time delays (*T*) allows each neurons to have access to *n* input values, corresponding to different input array instantaneous responses x(t−nT),⋯,x(t). The symbol σ(.) is the activation function in the hidden layer. In this case, we used the rectified linear unit (ReLU) activation function in the first layers (i.e., input and hidden layers), whereas the linear activation function was used in the output layer. Once a training dataset, {x,y}, with reference inputs, x, and their corresponding outputs, y, was provided, optimized weights, w, could be found by minimizing the cost function:

(3) $$E=\sum_{n}{\left(f\left(x_{n},w\right)-y_{n}\right)}^{2}$$

where $f(x_{n},w)$ is the output of the NN from the training inputs $x_{n}$. The optimization was done through stochastic gradient descent. This sub-step is called model training. In the next sub-step, the model was also evaluated to observe if the model specification was satisfactory. This step was done iteratively through k-fold cross-validation, which is a resampling technique designed to partition dataset into k (k-fold) subsets of data where one sample of them is held out while the model is trained with the remaining samples and then tested on the hold-outs. Iteration is also carried out to find the best model configurations, by adjusting the number of neurons for the input and hidden layers, weight initiation, the number of training cycles (epochs), and the learning rate.

Once the model met a satisfactory performance defined by a modeler, the testing data could be fed into the trained network to estimate PN concentration. The results were then evaluated through several performance metrics, which will be explained in the following sub-section.

### 3.3. Performance Metrics

In order to evaluate the best PN model through sensitivity analysis, we resorted to three metrics, as shown in Table 1. The symbols *y*, $\bar{y}$, and $\hat{y}$ represent the real measurement value, the mean of the measurement data points, and the estimated model value, respectively. The point number and the total estimated values from the models are indicated by the notations *i* and *n*, respectively. The coefficient of determination (R${}^{2}$) provides a measure of how well the observed outcomes are replicated by the model, based on the proportion of total variation of outcomes explained by the model. The mean absolute error (MAE) gives a simple interpretation as the average absolute difference between the predicted model values ($\hat{y}$) and the real measurement data points (y). Root mean squared error (RMSE) represents the standard deviation of the estimated errors (i.e., error residuals).

## 4. Results

### 4.1. Data Analysis

**Figure 6:**
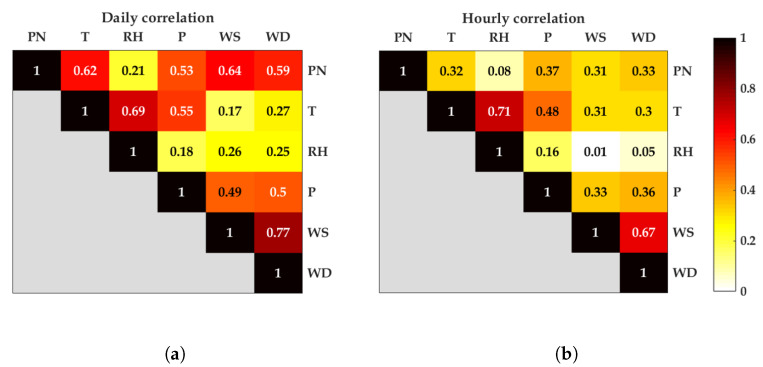
Matrix plots: absolute Pearson correlation coefficients between measured variables for daily and hourly averaged data. (**a**) Daily. (**b**) Hourly.

Figure 6 shows two matrix plots indicating the level of absolute Pearson correlation coefficients (PCC) between measured variables for daily and hourly data. The color closest to light yellow indicates a weak correlation, whereas the color closest to black indicates a strong correlation. It can be seen that the daily RH had a modest correlation with PN (PCC was about 0.21, with a *p*-value equal to zero), whereas the remaining daily measured variables had PCC values greater than 0.5, which indicated good correlations with PN. The hourly PCC values seemed to be reduced when compared to daily average data. The hourly RH showed a very weak correlation with PN (with PCC lower than 0.1, with a *p*-value equal to 0.54). Other meteorological variables, such as T, P, WS, and WD, still demonstrated satisfactory correlation with PN, ranging between 0.31 and 0.37 (with a *p*-value equal to zero).

**Figure 7:**
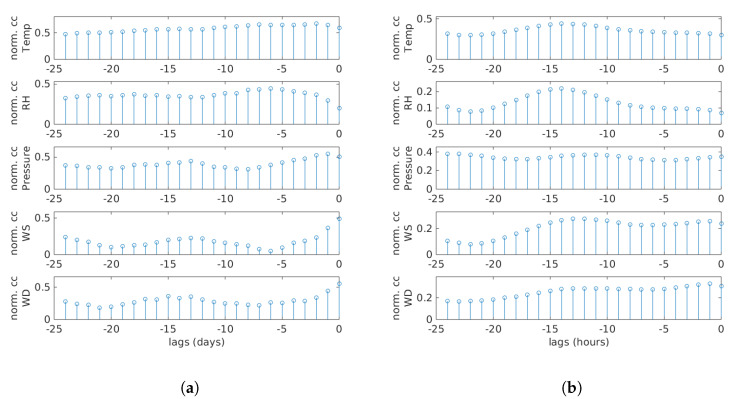
Cross-correlation between PN data and meteorological parameters for daily and hourly averaged data. Different time lags are shown on the x-axis, whereas the y-axis represents normalized correlation coefficients (norm. cc). (**a**) Daily. (**b**) Hourly.

Figure 7 shows the cross-correlation between PN and meteorological variables for daily (Figure 7a) and hourly (Figure 7b) averaged data. The x-axis shows different time lags, and the y-axis represents normalized correlation coefficients (abbreviated as norm. cc in the Figure). Both sub-figures demonstrate clearly that previous meteorological variables influenced the current PN concentration. Therefore, the use of time-delayed meteorological measurements may be beneficial in improving PN modeling accuracy based on the hourly data.

In general, the use of a large number of inputs typically increases the model complexity, leading to limited model performance. On the other hand, limiting the number of inputs also allows a model to be used without depending on many other measurements in practice. Therefore, it is vital to consider these effects when determining the number of inputs involved in modeling [23]. Since the matrix plotted only indicated bivariate correlation analysis, i.e., the correlation between two variables, sensitivity analysis was a useful method to investigate the multivariate measured variables influencing PN concentration. Sensitivity analysis was performed by training and testing PN modeling on all possible measured variable combinations, then the best combination of measured variables explaining PN concentration could be used as a final PN model.

### 4.2. Sensitivity Analysis

We resorted to two different types of ANN, called FFNN and TDNN, with the model specifications mentioned in Section 3. These models were then tested on all combination indexes of measured meteorological variables to perform sensitivity analysis, as illustrated in Figure 4. The models were trained and tested twice using daily and hourly average data. In this way, several best combinations of meteorological variables could be evaluated, and then, the best combination would be selected to be used as the inputs of PN models. The number of combinations of the variables used was 5 (if one variable was used), 10 (if two variables were used), 10 (if three variables were used), 5 (if four were variables used), and 1 (if five variables were used), with the total combinations being 31.

**Figure 8:**
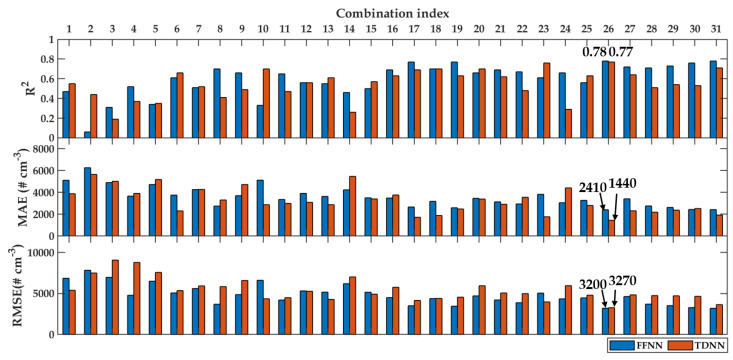
Performance metrics of daily modeling using FFNN (blue) and TDNN (red). The top, middle, and bottom sub-figures are R${}^{2}$, MAE, and RMSE, respectively.

**Figure 9:**
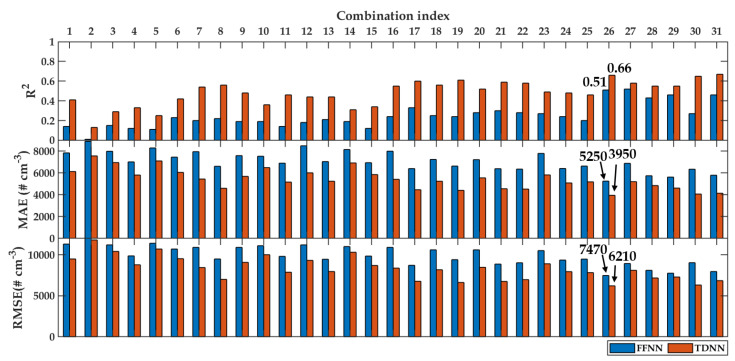
Performance metrics of hourly modeling using FFNN (blue) and TDNN (red). The top, middle, and bottom sub-figures are R${}^{2}$, MAE, and RMSE, respectively.

The performance metrics of modeling using these input variable combinations were tested according to R${}^{2}$, MAE, and RMSE. Figure 8 and Figure 9 present the performance metrics of PN modeling for daily and hourly averaged data, respectively. The blue bars are FFNN, whereas the red bars are TDNN. The low values of MAE and RMSE indicated that the models’ performance was better than the high values of these metrics. On the other hand, the high R${}^{2}$ values indicated that the models’ performance was better than the lower values. Since there were three metrics involved, the first priority was given to R${}^{2}$, then MAE and RMSE.

It can be seen that for both models, i.e., FFNN and TDNN, applied on both types of data averaging, having much fewer inputs did not provide adequate model performance because the inputs used were not informative enough to describe the PN concentration. As a general rule, having more variables for the inputs increases modeling accuracy.

Through the evaluation of the R${}^{2}$ values, Figure 8 (daily data averaging) shows that both models performed well when the models used at least four measured variable combinations. From these, the best R${}^{2}$ values were found at the combination indexes of 26 and 31. R${}^{2}$ values for FFNN were found to be the same for both models, that is equal to 0.78. However, the R${}^{2}$ value for TDNN for the combination index 26 (R${}^{2}$ = 0.77) was better than Number 31 (R${}^{2}$ = 0.71). Therefore, we decided to use Combination Index 26 for daily PN modeling with T, RH, P, and WS as input variables. In particular, FFNN seemed to be better than TDNN by observing R${}^{2}$ and RMSE values. Although MAE showed otherwise, we decided to resort to FFNN as the PN model because of the simplicity of model’s specification, development, and usage. Overall, when there were more than four inputs involved, FFNN also provided better performance than TDNN.

On the other hand, Figure 9 demonstrates clearly that TDNN was better than FFNN in all performance metrics across all input combinations. As in the case of daily data averaging, the best two candidates were found in the combination indexes 26 and 31. However, the R${}^{2}$ value of the combination index 31 ($R^{2}=0.67$) was slightly better than 26 ($R^{2}=0.66$). In this case, we decided to use the combination index 26 due to several reasons. First, the combination index 26 was found to be in agreement with the modeling using daily averaged data. Second, the combination index 26 had the best performance in terms of the MAE and RMSE metrics. Third, the best performing model 26 excluded the WD variable. WD was a circular variable, and in this study, we showed it in the scale of 0${}^{{}^{\circ}}$ to 360${}^{{}^{\circ}}$, which created discontinuity at the north. To tackle this, a trigonometric function had to be applied to resolve WD into two perpendicular directions before the data analysis. Finally, it was better to have a model that used fewer input variables if the performance was similar to a model with additional inputs. Therefore, in practice, the model with fewer inputs relied on fewer measurements (i.e., instruments). In summary, both models (using daily and hourly averaged data) used the combination index 26 with measured variables of T, RH, P, and WS. For now on, we present the results of PN models using the combination index 26.

**Figure 10:**
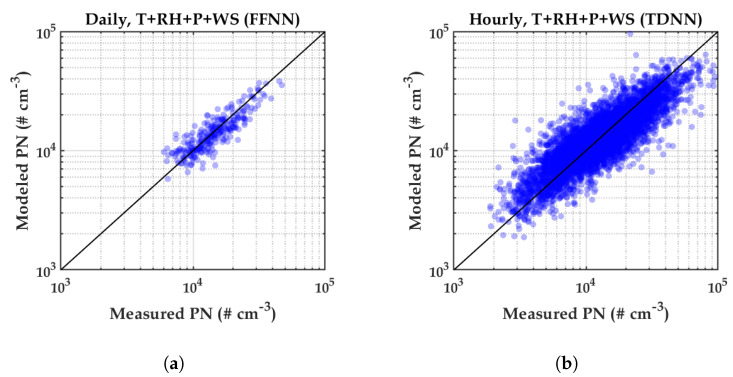
Scatter plot between PN measurement and PN estimation using four measured meteorological variables (T, RH, P, and WS). (**a**) Daily. (**b**) Hourly.

**Figure 11:**
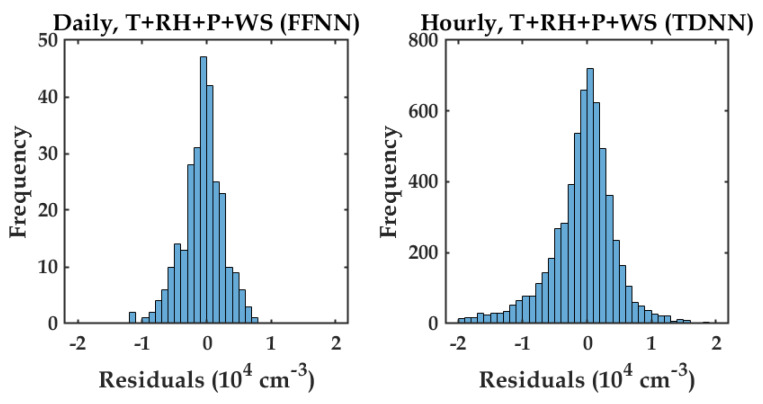
Histograms of residual error between the reference instrument and PN estimation using four measured variables (T, RH, P, and WS).

Figure 10 shows the scatter plots between the reference measured PN and the modeled PN using variables T, RH, P, and WS, for daily (Figure 10a) and hourly (Figure 10b) averaged data. It can be seen that most estimated data points of PN concentration for both averaged data followed the 1:1 line. Figure 11 shows the histograms of residual error between the PN measurement and the PN model for daily (left) and hourly (right). The figure shows that the majority of residual data points lied around zero residual error. This indicated that most of the estimated PN data points were precise.

**Figure 12:**
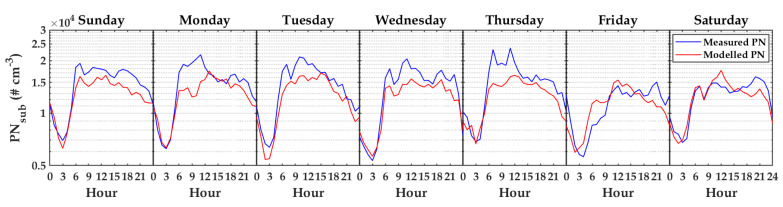
The median of diurnal cycles calculated on different days for measured PN and modeled PN (No. 26).

**Figure 13:**
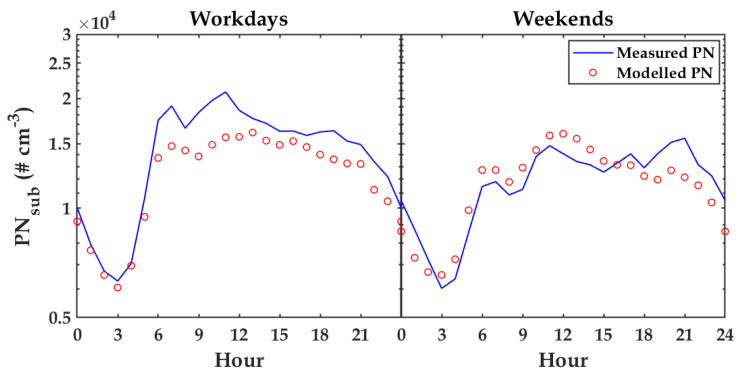
The median of diurnal cycles calculated on workdays and weekends for measured PN and modeled PN (No. 26).

Figure 12 presents the median of diurnal cycles calculated on all days in a week for measured PN (blue) and modeled PN (red). Furthermore, Figure 13 shows the median of diurnal cycles calculated on workdays and weekends for measured PN (blue) and modeled PN (red). Both figures show that the PN model followed the patterns of diurnal cycles in these two scenarios well. The results emphasized that PN modeling, using TDNN with input variables of T, RH, P, and WS, was reliable. Even though the variable RH presented a weak correlation to PN as shown in Figure 6a,b, through sensitivity analysis, it was found that it became a good feature when combined together with other variables. This indicated that using bivariate correlation analysis may not be optimal to find the best combination of variables to estimate PN concentration. Therefore, sensitivity analysis was a suitable method in this case.

## 5. Conclusions

Due to the adverse health effects on the human respiratory system, PN concentration is a vital air pollutant to be measured or estimated if the measurement is not available or there are massive missing data. This paper presented applying sensitivity analysis in the modeling framework of PN estimation. A feed-forward neural network (FFNN) and a time-delay neural network (TDNN) were chosen as the PN modeling tools. The sensitivity analysis utilized these models to be applied to different meteorological variables to find the best combinations as model inputs to estimate PN concentrations. In this case, sensitivity analysis was found to be more effective than bivariate correlation analysis. Through Pearson correlation coefficient (PCC) analysis, the RH variable was found to have a weak correlation with PN. However, when performing sensitivity analysis, RH was included in the top four best variables for PN modeling. The best combination of measured variables was T, RH, P, and WS. Using these variables as the model inputs, the best modeling techniques were FFNN for daily averaged data with R${}^{2}=0.77$ and TDNN for hourly averaged data with R${}^{2}=0.66$. The hourly estimation also followed the patterns of the diurnal cycle well, indicating that the established PN model was promising with a satisfactory accuracy.

Nevertheless, this method would be less effective and efficient once the number of measured variables and the amount of datasets become massive. For example, this might take place when the datasets are comprised of more than ten variables or the measurement data resolution is in the scale of minutes for a year-long dataset. Consequently, the method’s implementation would be computationally demanding. One solution is to use extra computational resources to parallelize the algorithms, such as through deployment on a computer cluster. Alternative solutions are to implement automatic input selection as proposed in [23] or to apply methods that are capable of performing variable selection, such as LASSO (least absolute shrinkage and selection operator) [42], which have also been used in air pollutant monitoring [43]. Furthermore, since the developed methods were based on statistical methods that utilized data from a specific region, they might work very well in the training location or in areas with similar emissions, processes, and meteorological conditions. The developed models could be generalized by training them using data measured from different areas. The transfer learning mechanism could be applied to re-train the pre-trained models once there new measurement data are received from the same or a different area [44].

Future works include the use of more time-series models to accommodate time-dependent variables in the form of white-box models, such as dynamic models or black-box models, such as long short-term memory (LSTM). Additional experiments will also be done to include the measurements of trace gases and radiation variables, which might also impact and improve the estimation of PN concentration. Finally, in order to establish a spatio-temporal PN map, more measurements at different locations are needed, and the missing measurements can be done through models’ interpolation.

## Figures and Tables

**Table 1:** The performance metrics used in the sensitivity analysis for the PN models’ evaluation.

Performance Metrics	Formulation
Coefficient of Determination	$R^{2}=1-\frac{\sum_{i=1}^{n}{\left({\hat{y}}_{i}-y_{i}\right)}^{2}}{\sum_{i=1}^{n}{\left(y_{i}-\bar{y}\right)}^{2}}$
Mean Absolute Error	$\mathrm{MAE}=\frac{\sum_{i=1}^{n}\left|{\hat{y}}_{i}-y_{i}\right|}{n}$
Root Mean Squared Error	$\mathrm{RMSE}=\sqrt{\frac{\sum_{i=1}^{n}{\left({\hat{y}}_{i}-y_{i}\right)}^{2}}{n}}$

## Abbreviations

The following abbreviations are used in this manuscript:

ANN	Artificial neural network
CMAQ	Community multiscale air quality
CO	Carbon monoxide
CPC	Condensation particle counter
FFNN	Feed-forward neural network
LASSO	Least absolute shrinkage and selection operator
LSTM	Long short-term memory
MAE	Mean absolute error
MENA	Middle East and North Africa
NNs	Neural networks
NO${}_{2}$	Nitrogen dioxide
O${}_{3}$	Ozone
OPS	Optical particle sizer
P	Absolute pressure
PCC	Pearson correlation coefficients
PM	Particulate matter
PM${}_{10}$	Particulate matter smaller than 10 μm
PM${}_{2.5}$	Particulate matter smaller than 2.5 μm
PN	Particle number
R${}^{2}$	Coefficient of determination
ReLU	Rectified linear unit
RF	Precipitation
RH	Relative humidity
RMSE	Root mean squared error
RNN	Recurrent neural network
SMPS	Scanning Mobility Particle Sizer
SO${}_{2}$	Sulfur dioxide
T	Temperature
TDNN	Time-delay neural network
UAM	Urban airshed model
UFPs	Ultra-fine particles
WD	Wind direction
WHO	World Health Organization
WS	Wind speed
